# Manual of Chemical Analysis as Applied to Examination of Medicinal Chemicals

**Published:** 1873-09

**Authors:** 


*Manual of Chemical Analysis as applied to The Examination of Medicinal Chemicals. A Guide for the determination of their Identity and Quality, and for the detection of Impurities and Adulterations.* By Frederick Hoffman, Ph. D. New York: D. Appleton & Co., 1873. Buffalo: Martin Taylor.

This is a work which will be of great value to Druggists and others in the habit of dealing with Medicinal Chemicals. The directions for ascertaining their identity and purity are well given and are such as can be easily applied by the majority of those wishing to use them. The work is amply illustrated, most of the apparatus used being figured in its appropriate place. As a work of reference for pharmaceutists and pharmaceutical students, it merits their careful attention.

The work consists of some three hundred and eighty pages and is printed in clear plain type, handsomly bound in cloth.

